# The escalating health crisis in Gaza amidst armed conflict and heatwaves

**DOI:** 10.1080/16549716.2025.2513856

**Published:** 2025-06-10

**Authors:** Luc Souilla, Amira Shaheen, Amira N. Mostafa, Samer Abuzerr

**Affiliations:** a Montreal Heart Institute and School of Kinesiology and Exercise Science, Université de Montréal, Montréal, Quebec, Canada; b Centre on Climate Change and Planetary Health, London School of Hygiene and Tropical Medicine, London, UK; c Public Health Department, Faculty of Medicine and Health Sciences, An-Najah National University, Nablus, Palestine; d Technical Office, Egyptian Meteorological Authority, Cairo, Egypt; e Astronomy, Space Science and Meteorology Department, Faculty of Science, Cairo University, Giza, Egypt; f Department of Medical Sciences, University College of Science and Technology, Khan Younis, Gaza, Palestine

**Keywords:** Palestine, vulnerability, heat stress, humanitarian crisis, climate change

## Abstract

The ongoing conflict in Gaza has resulted in a catastrophic humanitarian crisis, displacing over 1.7 million people and causing widespread damage to infrastructure, which has severely limited access to adequate shelter, clean water, and healthcare. With summer temperatures exceeding 38°C (100 °F), conflict has heightened the population’s vulnerability to heat-related health risks, compounded by a combination of environmental, individual, and conflict-induced factors. Gaza’s dense population, high numbers of vulnerable groups (e.g. infants, pregnant women, the elderly), and widespread pre-existing health conditions further amplify susceptibility to heat stress. Overcrowded shelters foster rapid dehydration and the spread of infectious diseases, while the destruction of Gaza’s power grid has led to widespread electricity shortages, depriving families of fans, air conditioning, and refrigeration, which are critical for cooling. Immediate global intervention is required to implement emergency public health measures and establish long-term resilience to extreme heat. Proposed actions include the provision of solar-powered cooling shelters, ensuring access to clean drinking water, distributing essential supplies such as solar-powered fans and hydration kits, and investing in climate-resilient infrastructure. Without urgent action, the convergence of extreme heat and the ongoing conflict in Gaza threatens to trigger a devastating heat-related health crisis that disproportionately affects the most vulnerable segments of the population.

## PAPER CONTEXT

- **Main findings** Rising temperatures in Gaza, worsened by climate change and conflict, can significantly increase heat-related health risks, especially among vulnerable populations.
- **Added knowledge** This article emphasizes how the interplay of environmental degradation, violence, and a collapsing healthcare system amplifies the threat of extreme heat in Gaza.
- **Global health impacts for policy and action**Immediate preventive measures and long-term strategies are essential to mitigate these risks and enhance a region’s resilience to extreme heat.

## Background

The Gaza Strip has long been marked by cycles of conflict, political instability, and humanitarian crises. The most recent war on Gaza, which began on 7 October 2023, has resulted in widespread destruction, mass displacement, and near-total collapse of essential infrastructure, including the healthcare system. While global attention has largely focused on political ramifications and the mounting death toll, an equally critical yet under-recognized threat looms: the intensifying environmental stress caused by extreme heat and recurrent heatwaves. Climate-adaptative public health measures should be treated as essential components of humanitarian aid, alongside food, shelter, and medical care.

This article explores the synergistic effects of heatwaves and conflict on population health in Gaza, emphasizing how environmental vulnerability and systemic fragility intersect with increasing human suffering. It calls for urgent, coordinated public health interventions to address this escalating crisis.

## Characterizing heat intensity in the Gaza Strip

Heat-health risks emerge from the interaction of environmental hazards (e.g. high temperatures), population exposure, underlying vulnerabilities, and poor adaptative capacity of the community [1]. Extreme heat events – commonly referred to as ‘silent killers’ – are becoming an alarming reality across the Arab region, where some areas routinely exceed 50°C in summer and face chronic water scarcity. This trend is especially concerning in the world’s most water-stressed region [2]. In Gaza, the impacts of climate change are acutely felt. Between 1977 and 2020, the average and maximum temperature in the Strip increased by two degrees, with the steepest rise occurring after 1990 [3–5]. From April through October, temperatures frequently surpass 38°C in the shade, occasionally spiking to 50°C under direct sun exposure during summer heatwaves [6]. Climate models project that, under a high-emission scenario, mean annual temperatures in the occupied Palestinian territory could rise by as much as 4.4°C by the end of the century [7].

Heatwaves, defined as prolonged periods of abnormally hot weather relative to a specific temperature threshold, are becoming more frequent and intense [8]. Between 1981 and 2010, approximately 15% of the days met this threshold, with heatwaves occurring more frequently in Gaza in recent years [9]. Projections suggest that by 2100, this could increase to 60% under a high-emission scenario and 25% under a low-emission scenario [10]. One study found that Gaza experiences an average of 31.3 weeks per year with weekly temperatures exceeding 19°C – levels associated with notable public health risks [11]. In April 2024, a poorly reported and under-investigated heatwave witnessed temperatures reaching 40°C–13°C above the seasonal average – and resulted in the death of at least two children [12].

This tragic event underscores the broader environmental and hazard-related threats confronting Gaza. The geographic and climatic profile of the region makes it particularly susceptible to compound risks, where extreme heat interacts with other environmental stressors, such as high humidity, droughts, and air pollution. These risks are further exacerbated by ongoing conflicts. Recent bombardments have reportedly produced over 50 million tons of debris and greenhouse gas emissions comparable to those of entire climate-vulnerable countries, severely degrading air, soil, and water quality but also deforestation and loss of biodiversity [13–15].

The convergence of natural and conflict-driven environmental hazards in Gaza creates a uniquely complex and dangerous public health zone [16]. While high temperatures also affect other countries in the region, Gaza’s humanitarian crisis – including widespread displacement, limited infrastructure, and collapse of basic services – substantially magnifies the population’s vulnerability and severely restricts their adaptive capacity. Despite these realities, there remains a striking lack of research on the health effects of extreme heat in Gaza, emphasizing the urgent need for targeted context-specific studies to inform evidence-based interventions in one of the world’s most climate-vulnerable and conflict-affected populations.

## What makes Gaza citizens more susceptible to heat?

The conflict in Gaza has intensified the population’s vulnerability to extreme heat, driven by economical struggles, and demographic pressure, environmental degradation, and health risks [17]. Spanning just 365 square kilometres, the Gaza Strip houses over two million people, making it one of the world’s most densely populated regions. Military bombardment has damaged over 92% of housing units, forcing most of the population to live in hazardous conditions [18]. Over 90% of Gaza’s residents are displaced, sheltering in overcrowded tents or damaged buildings lacking insulation, ventilation, and sun protection [19]. These conditions are especially dangerous during extreme heat.

Access to clean water, a critical defence against heat stress, is severely limited. Since the conflict began, only one in three people have received the minimum recommended daily water intake [18], which becomes life-threatening at high temperatures. Electricity infrastructure has been decimated, severely limiting access to cooling methods, such as fans or refrigeration. Power outages, combined with restricted access to fuel and functional electrical systems, have rendered basic heat-cooling strategies ineffective. In many shelters, available space per person is just 1.5 square meters – half the minimum emergency standard – creating stifling indoor environments [18].

Gaza’s population structure also intensifies vulnerability: approximately 47% of the population are under 15 years old, 3.0% are elderly, and 2.5% are pregnant women – with an average of 130 births occurring daily [20,21]. These groups are biologically more susceptible to heat-related stress due to reduced thermoregulatory capacity, including limited ability to sweat or adjust behaviour to avoid heat [22–24]. Physiologically, the human body relies on cardiovascular and thermoregulatory systems – such as sweating and increased blood circulation – to maintain a stable core temperature [25]. However, in Gaza, widespread chronic conditions such as hypertension, diabetes, and cardiovascular disease significantly impair these mechanisms [26,27]. These diseases limit the body’s ability to dissipate heat, increasing susceptibility to heat exhaustion, heatstroke, and cardiovascular events [28]. Notably, ischemic heart disease was the leading cause of death in Palestine in 2023 [20], a statistic expected to worsen under extreme heat.

Further compounding risk is the estimated 116,000 individuals with war-related injuries [29], especially children, which can have diminished physiological resilience and capacity to engage in adaptive behaviors such as hydrating regularly or moving to cooler environments. The spread of infectious diseases has surged during the conflict, worsening public health [30,31]. Diarrheal diseases are strongly correlated with rising temperatures and contribute to elevated core body temperatures and severe dehydration – especially dangerous for children and infants [11,32]. During summer, these risks intensify as poor sanitation and limited medical care fuel outbreaks.

Overlaying these risks is Gaza’s collapse of the health care system. According to the World Health Organization, as of April 2025, there have been over 671 attacks on health infrastructure, personnel, and supplies [33]. Fewer than half of Gaza’s hospitals partially function, and approximately 12,000 people need urgent medical attention [34]. The looming summer heat will place further pressure on this already overwhelmed system, increasing the risk of avoidable mortality due to heat-related illnesses.

**Figure 1.**
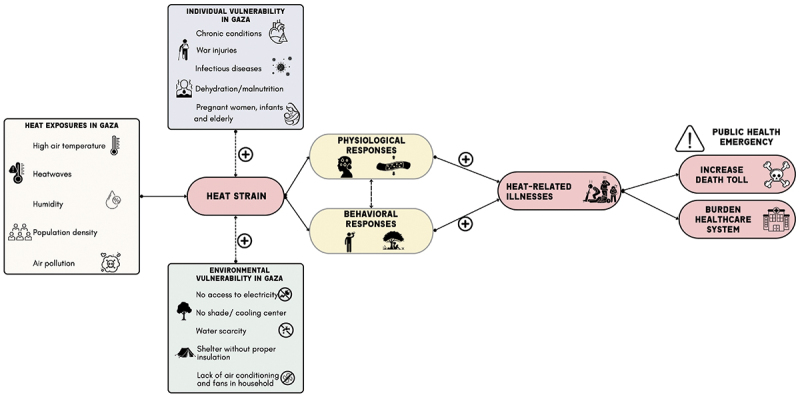
Potential multifaceted challenges faced by Gazans during the extreme heat events or high temperature.

This confluence of environmental, medical, and infrastructural vulnerabilities makes Gaza’s population among the most susceptible to extreme heat impacts globally (Figure 1). The situation demands immediate international attention and coordinated action to mitigate the deadly effects of extreme temperatures in the coming months and prevent further humanitarian catastrophe.

## How can the potential health threats of high temperatures be prevented?

As summer approaches, the convergence of extreme heat and conflict-related hardships in Gaza presents a critical public health emergency. Last year’s record-breaking temperatures in the region – such as Tel Aviv’s 40°C in April – serve as an alarming indicator of what lies ahead [12]. Normally, individuals could rely on adaptive strategies, including staying in air-conditioned spaces, using fans, accessing shaded areas, remaining indoors during peak heat, and staying hydrated with cool drinking water [35]. However, these basic coping mechanisms are largely inaccessible in Gaza due to ongoing conflict, displacement, infrastructure collapse, and severe resource shortages.

The implementation of conventional public health measures – such as establishing community cooling centres, distributing public health messaging, and deploying emergency health teams – constrained by security issues and destroyed infrastructure. In this setting, heat-related illnesses and deaths threaten vulnerable groups.

Simultaneously, long-term strategies are vital for building resilience to extreme heat. These include investing in climate-adaptive infrastructure and integrating heat mitigation into the design of humanitarian aid programmes. Without immediate and sustained action, the compounded threats of high-temperature climate change and conflict will continue to endanger the lives of the people of Gaza.

Addressing these risks requires more than ending hostilities; it requires an urgent, coordinated humanitarian response focused on context-appropriate public health interventions. The World Health Organization should take the lead in coordinating health interventions, supporting hospitals with essential medical supplies, and helping restore critical health services. The United Nations High Commissioner for Refugees has an important role to play in responding to the needs of displaced families, by providing temporary shelter and protection from heat-related risks. The Palestinian Red Crescent Society must continue to play a vital role in delivering emergency care. Finally, local health ministries must be empowered to register people suffering from heat-health illnesses, manage logistics, and coordinate with international partners to ensure that interventions are effective and relevant. International agencies must prioritize preventive measures to mitigate heat impacts, especially for high-risk populations affected by the conflict, including the elderly, infants, pregnant women, those with chronic diseases, and injury recovery patients [36–39].

Immediate actions should include the following:

- Deployment of solar-powered cooling shelters in displacement camps and other high-density living areas.
- Guaranteed access to safe drinking water by scaling up emergency water trucking and desalination support.
- Distribution of emergency heat relief supplies, including solar-powered fans, reusable water bottles, oral rehydration salts, and hydration kits.
- Dissemination of heat-health protection guidelines adapted to the local context and disseminated through trusted community channels as well as by rebuilding a sustainable education system [40].

The effectiveness and sustainability of these measures necessitate the establishment of secure conditions through ceasefire, lifting blockades, and addressing widespread destruction.

Long-term resilience efforts must include integrating heat risk mitigation into humanitarian frameworks, investing in climate-resilient infrastructure, enhancing green spaces, and ensuring that shelter designs account for thermal protection. Without sustained action, the threats of climate change and conflict will continue to exacerbate suffering among the Gaza population. A proactive approach rooted in climate-adaptive public health strategies and humanitarian justice are essential to protect lives in this vulnerable region.

## Limitations

It is important to acknowledge that this article has several limitations due to the ongoing humanitarian crisis in Gaza. Data underreporting in conflict zones and restricted access to the field hinder a comprehensive understanding of the situation. These challenges not only result in incomplete or missing data but also prevent competent authorities from accurately documenting the number of individuals affected by heat-related illnesses and mortality in Gaza. However, most studies over Gaza depend on temperature data derived from remotely sensed data by weather satellites, as well as reanalysis models that generate meteorological datasets by combining observations into estimates using modelling and data assimilation. These remote sensing and reanalysis data partially compensate for lack of ground measurement, but with an uncertainty in accuracy that needs consideration [41,42].

## Conclusion

The compounding crises in Gaza, marked by conflict, environmental decline, and climate change impacts, have created extreme vulnerability. As heat events become more frequent and severe, their health consequences are amplified by displacement, inadequate shelters, collapsed services, and eroded coping mechanisms. Gaza’s and fragile context magnifies these heat-health risks, placing millions – especially children, the elderly, pregnant women, and people with chronic illnesses – at an immediate and life-threatening peril. This convergence of climate and humanitarian crises is not unique in the region, where similar dynamics can be observed in conflict-affected countries, such as Syria, Yemen, and Sudan.

Urgent international action is needed to recognize heat as a public health emergency in conflict zones. This includes deploying life-saving interventions, such as cooling systems, water provision, and heat relief supplies, alongside strategies that build climate resilience in humanitarian planning. Failure to respond will lead to preventable deaths and further entrench existing inequalities. Gaza’s population cannot afford to wait for political stability to confront climate crisis. Ignoring the climate-health nexus in Gaza today risks normalizing inaction in other conflict settings facing escalating climate threats.

## Data Availability

Data sharing is not applicable to this article as no data were created or analysed in this study.
